# Preoperative Gadoxetic-Acid-Enhanced MRI Features Associated with Rapid Recurrence (<6 Months) After Curative Resection for Hepatocellular Carcinoma

**DOI:** 10.3390/diagnostics16071108

**Published:** 2026-04-07

**Authors:** Jeong Woo Kim, Chang Hee Lee

**Affiliations:** Department of Radiology, Korea University Guro Hospital, Korea University College of Medicine, 148 Gurodong-ro, Guro-gu, Seoul 08380, Republic of Korea; pridebio@naver.com

**Keywords:** hepatocellular carcinoma, gadoxetic-acid-enhanced MRI, rapid recurrence

## Abstract

**Background/Objectives**: To evaluate the incidence of rapid recurrence within 6 months of curative resection for hepatocellular carcinoma (HCC) and to identify preoperative gadoxetic-acid-enhanced MRI features associated with rapid recurrence (<6 months) in the entire cohort. **Methods**: This retrospective study included 200 patients who underwent curative resection for HCC and had preoperative gadoxetic-acid-enhanced MRI between January 2016 and December 2023. Patients were categorized into a rapid recurrence group (*n* = 21) and a non-rapid recurrence group (*n* = 179). Preoperative MRI features, including tumor size, multiplicity, tumor margin, arterial peritumoral enhancement, peritumoral hepatobiliary phase (HBP) hypointensity, diffusion restriction, apparent diffusion coefficient (ADC) values, and presence of non-hypervascular hepatobiliary phase hypointense nodule (NHHN), were evaluated. Univariate and multivariate Firth penalized logistic regression analyses were performed. **Results**: Rapid recurrence occurred in 10.5% (21/200) of patients (median, 4.0 months). Multivariate analysis revealed that larger tumor size (odds ratio [OR], 1.25 per 1-cm increase; *p* = 0.012) and the presence of NHHN (OR, 11.30; *p* < 0.001) were independent predictors of rapid recurrence. A nomogram incorporating these features demonstrated excellent discriminative performance, with a bootstrap-corrected area under the curve (AUC) of 0.864 (95% CI, 0.791–0.922). **Conclusions**: The presence of NHHN and larger tumor size on preoperative MRI were associated with rapid recurrence (<6 months) after curative resection for HCC. These findings may provide additional support for preoperative risk stratification and the planning of postoperative surveillance strategies.

## 1. Introduction

Hepatocellular carcinoma (HCC) is one of the most common malignancies worldwide and remains a leading cause of cancer-related mortality [1,2,3]. Surgical resection is a potentially curative treatment for HCC in patients with preserved liver function. However, prognosis after hepatic resection remains poor due to a high recurrence rate, which is up to 70% at 5 years after surgery [2]. Postoperative recurrence of HCC is commonly classified based on the time of occurrence, as the underlying mechanisms and prognosis differ substantially [4,5]. Early recurrence, typically defined as recurrence occurring within 2 years of surgery, is generally considered to result from intrahepatic metastasis of the primary tumor and is associated with aggressive tumor biology, including microvascular invasion (MVI) and poor tumor differentiation. In contrast, late recurrence, occurring after 2 years, is more likely related to de novo multicentric hepatocarcinogenesis in the background of chronic liver disease [4,5]. Several previous studies have demonstrated that early recurrence is associated with significantly worse survival than late recurrence, emphasizing the importance of identifying patients at high risk for early recurrence [4,6].

Preoperative gadoxetic-acid-enhanced MRI has been shown to provide imaging biomarkers predictive of early recurrence, including non-smooth tumor margin, peritumoral arterial enhancement, peritumoral hepatobiliary phase (HBP) hypointensity, and diffusion restriction [7,8,9,10]. In addition, several studies have shown that non-hypervascular hepatobiliary phase hypointense nodules (NHHNs) detected on preoperative MRI are associated with postoperative recurrence [11,12].

In clinical practice, a distinct subset of patients who develop very rapid recurrence within a few months of curative resection is occasionally encountered, which is often characterized by multifocal or disseminated appearance and extremely poor prognosis [13,14,15]. Previous studies have referred to this phenomenon in various terms such as early explosive recurrence or early disseminated recurrence, typically occurring within 3–6 months of surgery and associated with aggressive clinicopathologic features and poor outcomes [13,14,15]. However, these previous studies primarily focused on pathological or serologic risk factors, with limited research on preoperative imaging predictors. Additionally, specific preoperative gadoxetic-acid-enhanced MRI features of rapid recurrence (<6 months) have not yet been fully elucidated.

Therefore, the purpose of this study was (1) to evaluate the incidence of rapid recurrence within 6 months of curative resection for HCC, and (2) to investigate preoperative gadoxetic-acid-enhanced MRI features associated with rapid recurrence in the entire cohort.

## 2. Materials and Methods

### 2.1. Patients

**Figure 1:**
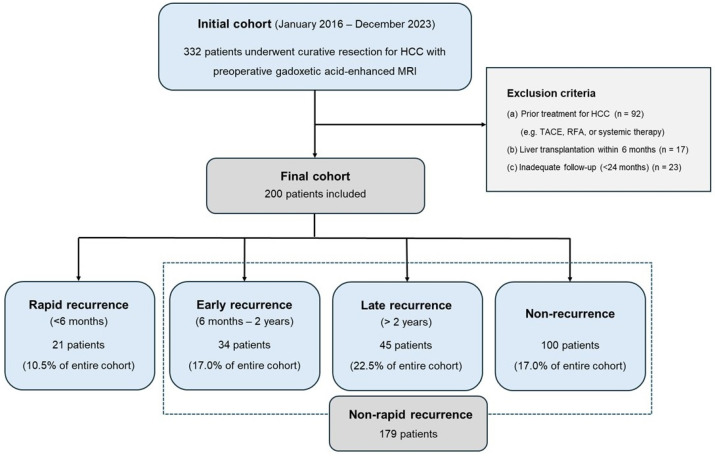
Flow diagram of the study population.

This retrospective study was approved by our institutional review board and the requirement for informed consent was waived. From January 2016 to December 2023, consecutive patients who underwent curative surgical resection for HCC at our institution were initially screened. The inclusion criteria were as follows: (1) Patients who underwent preoperative gadoxetic-acid-enhanced liver MRI within one month of surgery and (2) those who underwent curative resection (R0), defined as complete removal of the tumor with a tumor-free margin on pathological examination. Patients were excluded if they met any of the following criteria: (1) history of any prior treatment for HCC (e.g., TACE, RFA, or systemic therapy); (2) liver transplantation performed within 6 months of surgery; and (3) insufficient follow-up data or loss to follow-up within 24 months of surgery (except for those who developed recurrence within that period) (Figure 1).

Postoperative recurrence was defined as the appearance of newly developed lesions showing typical imaging features of HCC on follow-up dynamic CT or MRI. Patients were classified into four groups according to the time to recurrence after surgery: (1) rapid recurrence group (<6 months); (2) early recurrence group (6 months–2 years); (3) late recurrence group (>2 years); and (4) non-recurrence group (no recurrence for at least 2 years of the follow-up). The incidence of each group was calculated across the entire cohort.

Clinical data including age, sex, etiology (HBV, HCV, and others), cirrhosis, and tumor markers (AFP and PIVKA-II) were collected. Postoperative pathologic data including MVI and histologic tumor grade according to the Edmondson–Steiner classification were also obtained (Table 1).

### 2.2. MRI Acquisition

All MRI examinations were performed using a 3.0-Tesla system (Skyra; Siemens Healthineers, Erlangen, Germany) with a standard 18-channel body matrix coil and a table-mounted 32-channel spine matrix coil. The MRI protocol included unenhanced T1-weighted imaging (in-phase and opposed-phase), T2-weighted imaging, diffusion-weighted imaging (DWI), and dynamic contrast-enhanced imaging.

For dynamic contrast-enhanced study, gadoxetic acid (Primovist; Bayer Schering Pharma, Berlin, Germany) was administered as a bolus at a dose of 0.025 mmol/kg of body weight (0.1 mL/kg) and a flow rate of 1.0 mL/s, followed by a 20-mL saline flush. Dynamic images were obtained before contrast injection (pre-contrast) and at the arterial phase (20–35 s), portal venous phase (70 s), and transitional phase (2 min) using a three-dimensional (3D) T1-weighted gradient echo-sequence (VIBE). The HBP was acquired 20 min after the administration of the contrast agent using the same 3D VIBE sequence.

DWI was performed in the axial plane using a single-shot echo-planar imaging sequence with b-values of 0, 50, and 800 s/mm^2^, and apparent diffusion coefficient (ADC) maps were automatically generated.

The detailed MRI sequence parameters used in this study are summarized in Table 2.

### 2.3. Imaging Analysis

Preoperative MRI features were retrospectively evaluated for the rapid (*n* = 21) and non-rapid (*n* = 179) recurrence groups. Two board-certified abdominal radiologists (10 and 30 years of experience, respectively) independently reviewed the images. To minimize bias, the reviewers were aware that all included patients developed postoperative recurrence but were blinded to the timing of recurrence, as well as to all clinical, laboratory, pathological, and postoperative outcome data. The following preoperative imaging features were evaluated: (1) tumor margin (smooth or non-smooth); (2) arterial peritumoral enhancement; (3) peritumoral HBP hypointensity; (4) diffusion restriction; and (5) presence of NHHN. NHHN was defined as a distinct nodule showing HBP hypointensity without corresponding arterial phase hyperenhancement. Any discrepancies in imaging interpretation between the two radiologists were resolved by consensus review.

For quantitative analysis, tumor size and ADC values were independently measured by the two radiologists. To ensure measurement stability, each radiologist performed three independent measurements for each parameter, and their respective mean values were calculated. The final value used for statistical analysis was the average of the mean values obtained by the two radiologists. For ADC measurement, circular regions of interest were placed to cover the solid portion of the tumor while avoiding necrotic or hemorrhagic areas.

### 2.4. Statistical Analysis

Continuous variables were expressed as mean with standard deviation, and categorical variables were expressed as number (percentage). Comparisons between the rapid and non-rapid recurrence groups were performed using the Mann–Whitney U test for continuous variables and Fisher’s exact test for categorical variables.

Interobserver agreement for qualitative features was assessed using Cohen’s kappa statistics. The kappa (κ) values were interpreted as: <0.20, poor; 0.21–0.40, fair; 0.41–0.60, moderate; 0.61–0.80, substantial; and 0.81–1.00, almost perfect agreement. For quantitative variables, the intraclass correlation coefficient (ICC) was calculated based on the independent measurements of the two readers.

To identify preoperative MRI features independently associated with rapid recurrence, Firth penalized logistic regression was performed to reduce small-sample bias and address potential sparse-data separation. Univariate Firth penalized logistic regression analyses were first conducted for all preoperative imaging variables, including tumor size, multiplicity, tumor margin, arterial peritumoral enhancement, peritumoral HBP hypointensity, diffusion restriction, ADC value, and the presence of NHHN. All variables with *p* < 0.2 in univariate analysis were considered for the multivariate Firth logistic regression model. Odds ratios (ORs) with 95% confidence intervals (CIs) were reported.

The discriminative performance of the multivariate model was evaluated using the area under the receiver operating characteristic curve (AUC). A nomogram was constructed based on the multivariate logistic regression model. The point-based scale in the nomogram was assigned to each predictor based on its regression coefficient. The diagnostic accuracy of the model was further assessed by calculating sensitivity, specificity, positive predictive value (PPV), and negative predictive value (NPV) at the optimal threshold determined by Youden’s index. To assess the agreement between the predicted probabilities and the actual observed outcomes, a calibration plot was generated. The calibration of the nomogram was further evaluated using the Hosmer–Lemeshow goodness-of-fit test, where a non-significant *p*-value (*p* > 0.05) indicates a well-calibrated model.

To ensure the robustness of our predictive model and to obtain optimism-corrected estimates, internal validation was performed using a bootstrapping procedure with 10,000 iterations. This approach was used to estimate the 95% confidence intervals (CIs) for the odds ratios (ORs) and to validate the model’s predictive performance metrics, including the area under the curve (AUC). All statistical analyses were conducted based on these bootstrap-corrected estimates to minimize potential overfitting and to provide reliable preoperative risk assessment.

All statistical analyses were performed using SPSS (version 26.0; IBM Corp., Armonk, NY, USA) for descriptive statistics and group comparisons, and R software (version 4.2.2; R Foundation for Statistical Computing, Vienna, Austria) with the logistf package for Firth’s penalized logistic regression. A *p*-value of <0.05 was considered to indicate a statistically significant difference.

## 3. Results

### 3.1. Patients

In the entire cohort of 200 patients, the incidence of rapid recurrence (<6 months) was 10.5% (21/200). The remaining 179 patients (89.5%) were categorized into the non-rapid recurrence group, which included patients with early recurrence (*n* = 34, 17.0%), late recurrence (*n* = 16, 8.0%), and no recurrence during the follow-up period (*n* = 129, 64.5%). The median time to recurrence in the rapid recurrence group was 4.0 months (IQR, 3.0–5.0 months).

The baseline clinical and pathological characteristics of the 200 patients are summarized in Table 1. When comparing the rapid recurrence group with the non-rapid recurrence group, the rapid recurrence group (<6 months) showed significantly higher PIVKA-II levels (*p* = 0.007) and larger tumor size. The presence of pathologically confirmed MVI was significantly more frequent in the rapid recurrence group than in the non-rapid recurrence group (61.9% vs. 11.7%, *p* < 0.001). Other factors, including age, sex, etiology, presence of cirrhosis, AFP levels, and tumor grade did not differ significantly between the two groups.

### 3.2. Comparison of Preoperative MRI Features

The interobserver agreement for preoperative MRI features was almost perfect. The κ value for the presence of NHHN was 0.920, and for other qualitative features, it ranged from 0.851 to 1.000 (margin, 0.917; arterial peritumoral enhancement, 0.851; peritumoral HBP hypointensity, 0.894; and diffusion restriction, 1.000). The ICCs for tumor size and ADC values were 0.998 and 0.977, respectively, indicating excellent reliability.

Comparisons of preoperative gadoxetic-acid-enhanced MRI features between the rapid and non-rapid recurrence groups are presented in Table 3.

Among preoperative MRI features, the presence of NHHN was also significantly more frequent in the rapid recurrence group than in the non-rapid recurrence group (66.7% vs. 15.6%; *p* < 0.001). Tumor size was also significantly larger in the rapid recurrence group than in the non-rapid recurrence group (5.9 ± 4.4 cm vs. 3.1 ± 2.2 cm; *p* < 0.001).

In contrast, other preoperative MRI features, including non-smooth tumor margin, arterial peritumoral enhancement, peritumoral HBP hypointensity, and diffusion restriction, did not differ significantly between the two groups. ADC values were also comparable between the two groups. Additionally, further analysis was conducted to evaluate the relationship between the presence of NHHN and pathological MVI. In the entire cohort, there was a significant correlation between the presence of NHHN and MVI (*p* < 0.001).

### 3.3. Firth Penalized Logistic Regression Analysis

Results of univariate and multivariate Firth penalized logistic regression analyses for rapid recurrence compared with non-rapid recurrence are shown in Table 4.

In the univariate analysis, larger tumor size (OR, 1.30 per 1-cm increase; 95% CI, 1.13–1.49; *p* < 0.001) and the presence of NHHN (OR, 10.79; 95% CI, 4.00–29.11; *p* < 0.001) were significantly associated with rapid recurrence. Arterial peritumoral enhancement also showed a potential association (*p* = 0.086) and was therefore included in the multivariate model as a candidate predictor based on a significance threshold of *p* < 0.1. Other preoperative MRI variables, including multiplicity, non-smooth margin, peritumoral HBP hypointensity, diffusion restriction, and ADC value, were not significantly associated with rapid recurrence.

**Figure 2:**
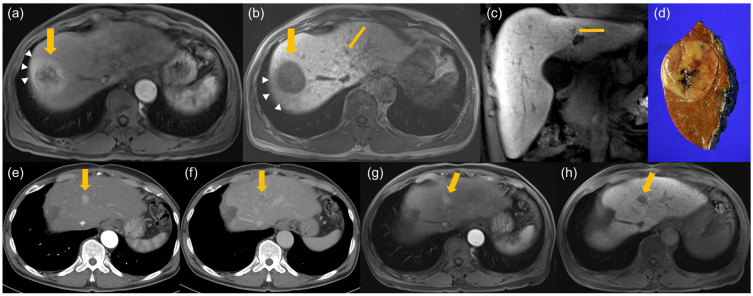
A patient with rapid recurrence (<6 months) after curative resection for hepatocellular carcinoma (HCC). (**a**–**c**) Preoperative gadoxetic-acid-enhanced liver MRI. (**a**) Arterial phase image shows a 4.3-cm hypervascular mass in segment 7/8 (thick yellow arrow) with peritumoral arterial enhancement (white arrowheads). (**b**,**c**) Axial and coronal hepatobiliary (HBP) images demonstrate the tumor as a hypointense mass (thick yellow arrow) with peritumoral HBP hypointensity (white arrowheads). Note a separate non-hypervascular hepatobiliary phase hypointense nodule (NHHN) in segment 4 (thin yellow arrow). (**d**) Photograph of the gross resected specimen confirming HCC. (**e**,**f**) Follow-up CT scans obtained 4 months after surgery. (**e**) Arterial phase and (**f**) delayed phase images reveal a newly developed 1.7-cm lesion showing arterial enhancement and delayed washout (yellow arrow) at the site corresponding to the preoperative NHHN. (**g**,**h**) Subsequent gadoxetic-acid-enhanced liver MRI confirms the recurrence (arrow). The previous NHHN has progressed into a hypervascular HCC, showing (**g**) arterial phase hyperenhancement and (**h**) an increase in size on the HBP image.

**Figure 3:**
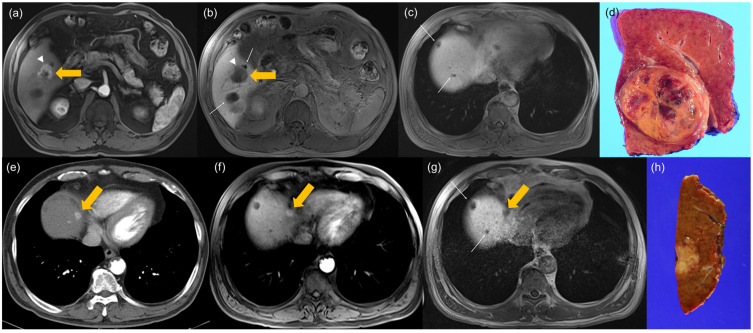
A patient with early recurrence (6 months–2 years) after curative resection for hepatocellular carcinoma (HCC). (**a**–**c**) Preoperative gadoxetic-acid-enhanced liver MRI. (**a**) Arterial phase image shows a 3-cm hypervascular mass (thick yellow arrow) with a non-smooth margin and peritumoral arterial enhancement (white arrowheads). (**b**,**c**) Axial hepatobiliary phase (HBP) images demonstrate the tumor as a hypointense mass (thick yellow arrow) with peritumoral HBP hypointensity (white arrowheads). Note several cysts in the liver (thin white arrows), but non-hypervascular hepatobiliary phase hypointense nodules (NHHNs) are not observed elsewhere in the liver, including liver dome. (**d**) Photograph of the gross resected specimen confirming HCC. (**e**) Arterial phase image of follow-up CT scan obtained 12 months after surgery reveals a new nodular enhancing lesion (yellow arrow) at the liver dome. (**f**,**g**) Subsequent gadoxetic-acid-enhanced liver MRI confirms the recurrence. The recurrent lesion (arrow) shows (**f**) arterial phase hyperenhancement and (**g**) hypointensity on the HBP image. (**h**) Photograph of the resected specimen from the second surgery confirming the recurrent HCC.

In multivariate analysis, larger tumor size (OR, 1.32 per 1-cm increase; 95% CI, 1.12–1.56; *p* = 0.001) and presence of NHHN (OR, 11.30; 95% CI, 3.75–34.04; *p* < 0.001) remained as independent preoperative predictors of rapid recurrence (Figure 2 and Figure 3).

### 3.4. Predictive Performance of the Model

**Figure 4:**
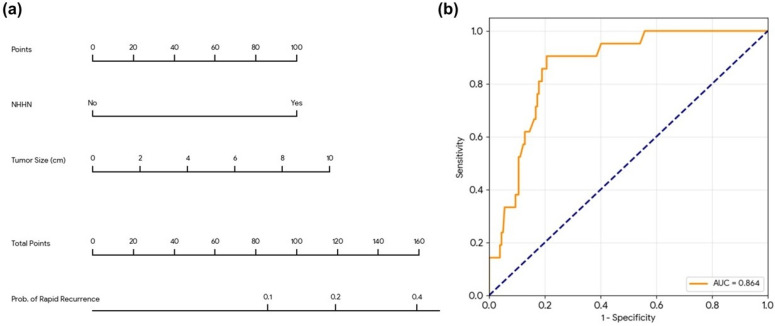
Development and validation of the predictive model for rapid recurrence. (**a**) Nomogram for predicting the individualized probability of rapid recurrence (<6 months) after curative resection. To use the nomogram, locate the patient’s tumor size and NHHN status, draw a line upward to the ‘Points’ axis to determine the score for each variable, and sum these scores to find the ‘Total Points,’ which corresponds to the risk of rapid recurrence at the bottom. (**b**) Receiver operating characteristic (ROC) curve of the combined model (Size + NHHN), showing an area under the curve (AUC) of 0.864.

To facilitate clinical application, a nomogram was developed based on the multivariate logistic regression model incorporating tumor size and the presence of NHHN (Figure 4a). The discriminative performance of this model was excellent, with an area under the curve (AUC) of 0.864 (95% CI, 0.791–0.922) (Figure 4b). At the optimal threshold, the model achieved a sensitivity of 90.5%, a specificity of 79.3%, a positive predictive value (PPV) of 33.9%, and a negative predictive value (NPV) of 98.6%.

The calibration of the nomogram was evaluated using the Hosmer–Lemeshow goodness-of-fit test, which yielded a *p*-value of 0.308, indicating that the model is well-calibrated and shows no significant lack of fit.

### 3.5. Prognostic Value of NHHN

**Figure 5:**
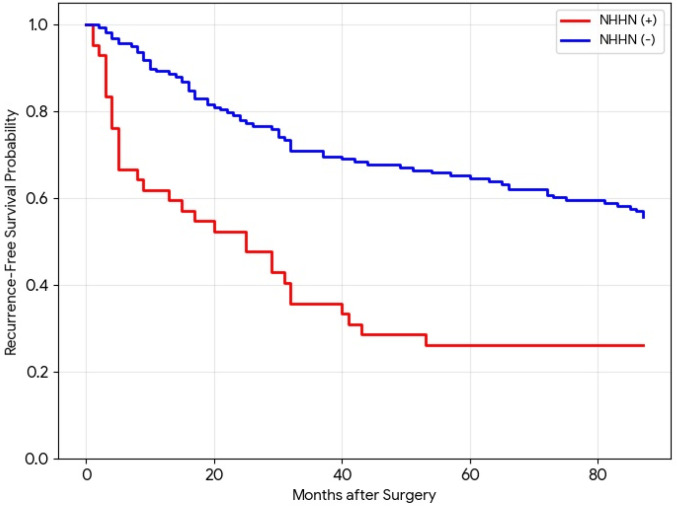
Kaplan–Meier curves for recurrence-free survival (RFS) according to the presence of non-hypervascular hepatobiliary phase hypointense nodules (NHHN) in the entire cohort (*n* = 200). Patients with preoperative NHHN (red line) show a significantly lower RFS rate compared to those without NHHN (blue line) (log-rank *p* < 0.001). The two curves diverge sharply within the first 6 months, with 6-month RFS rates of 66.7% for the NHHN-positive group and 95.6% for the NHHN-negative group, highlighting the clinical significance of NHHN in predicting rapid recurrence.

To evaluate the overall prognostic impact of NHHN, Kaplan–Meier analysis was performed for recurrence-free survival (RFS) across the entire cohort (*n* = 200). Patients with NHHN demonstrated significantly lower RFS rates compared to those without NHHN (Figure 5). Notably, the 6-month RFS rate was substantially lower in the NHHN-positive group than in the NHHN-negative group (66.7% vs. 95.6%). The survival curves showed a rapid divergence within the first 6 months, indicating that NHHN is a robust indicator of rapid recurrence in the clinical setting.

## 4. Discussion

In the present study, we investigated preoperative gadoxetic-acid-enhanced MRI features associated with rapid recurrence within 6 months of curative resection for HCC. Our results showed that approximately 10.5% (21/200) of the entire cohort experienced rapid recurrence. Notably, in the multivariate analysis, the presence of NHHN and larger tumor size were independently associated with rapid recurrence, whereas other preoperative MRI features previously reported to be associated with MVI and early recurrence did not show significant differences between the rapid and non-rapid groups.

Early recurrence of HCC after curative resection has been consistently associated with aggressive tumor biology [4,16]. Previous studies have consistently identified MVI and poor tumor differentiation as key pathological risk factors for early recurrence [6,17,18]. In addition, several previous studies have demonstrated that preoperative gadoxetic-acid-enhanced MRI features—such as non-smooth margin, arterial peritumoral enhancement, and peritumoral HBP hypointensity—are reliable imaging biomarkers for predicting MVI and subsequent early recurrence [7,8].

Interestingly, preoperative MRI features previously reported to be associated with MVI and early recurrence did not significantly differentiate patients with rapid recurrence (<6 months) in the entire cohort. Instead, the presence of NHHN and larger tumor size emerged as distinguishing features associated with rapid recurrence. The prognostic significance of NHHN in patients undergoing curative resection has been supported by previous studies [11,12,18]. In a prospective observational study, Toyoda et al. demonstrated that preoperative NHHNs detected on gadoxetic-acid-enhanced MRI were independently associated with increased recurrence after hepatectomy [11]. In that study, recurrence was mainly attributed to multicentric recurrence arising from pre-existing nodules, and serial imaging showed progression of NHHNs to hypervascular HCC during follow-up. Consistent with these findings, Lee et al. reported that the presence of NHHNs on preoperative gadoxetic-acid-enhanced MRI was a significant predictor of recurrence after both hepatic resection and radiofrequency ablation and could further inform treatment selection, highlighting their clinical relevance as imaging biomarkers of aggressive tumor biology [18]. A recent systematic review and meta-analysis also reported that the presence of NHHN was associated with a significantly increased risk of intrahepatic distant recurrence even in patients treated with curative hepatectomy [12]. These findings suggest that NHHNs may represent occult malignant or premalignant foci present at the time of surgery and provide important context for our observation that NHHNs are specifically associated with rapid recurrence.

One possible hypothesis to explain this immediate recurrence is the so-called “angiogenic switch” triggered by surgical stress. While the exact mechanism remains to be fully elucidated, previous experimental data have shown that liver resection induces a surge in growth factors, such as hepatocyte growth factor (HGF) and vascular endothelial growth factor (VEGF), which are involved in liver regeneration [19,20,21]. This pro-proliferative environment may potentially promote the progression of pre-existing lesions such as NHHNs. However, this proposed mechanism remains speculative, as our study does not include biomarker data to directly support this pathway. Therefore, this explanation should be interpreted with caution. Further experimental and longitudinal clinical studies incorporating imaging, molecular, and circulating biomarkers are required to validate this hypothesis.

The clinical utility of our findings lies in improving preoperative decision-making and postoperative surveillance strategies. Currently, major international guidelines classify nodules that do not meet the typical vascular criteria for HCC—such as NHHNs—as “indeterminate nodules” [2,22,23]. These guidelines generally recommend close radiological monitoring every 3 to 6 months due to their high potential for malignant transformation or the possibility of being early-stage HCCs. However, there is still no established consensus on how to manage these nodules identified concurrently with a primary HCC for which curative resection is planned. In patients undergoing curative resection for HCC, the concurrent presence of NHHNs on preoperative MRI may therefore influence preoperative decision-making, including surgical planning and the need for multidisciplinary discussion. Furthermore, these imaging findings may justify consideration of more intensive and individualized postoperative surveillance strategies, rather than standard follow-up protocols. Nevertheless, further prospective studies with large cohorts are warranted to clarify the optimal management of concomitant NHHNs and to establish evidence-based recommendations.

In our cohort, PIVKA-II was significantly associated with rapid recurrence in the univariate analysis (*p* = 0.003), reflecting its role as a marker for tumor aggressiveness. However, to maintain the primary focus of this study on preoperative imaging biomarkers, PIVKA-II was not included in the final multivariate model. This approach allowed us to specifically validate the independent predictive power of NHHN and tumor size, demonstrating that these MRI features provide sufficient and robust information for preoperative risk stratification even without the integration of serum biomarkers. To enhance the clinical utility of our findings, we developed a nomogram that allows for the preoperative estimation of rapid recurrence risk. By combining tumor size with NHHN, this tool offers a more comprehensive risk assessment than single-parameter approaches. Although our model showed excellent discriminative performance (AUC 0.843), the relatively small sample size necessitates caution in its interpretation. Further prospective multicenter studies are required to validate the generalizability of this nomogram before it can be widely implemented in clinical practice.

There are some limitations to this study. First, its retrospective nature and the relatively small sample size of the rapid recurrence group may limit generalizability. The number of events in the rapid recurrence group was relatively small, which resulted in wide confidence intervals for certain predictors, such as NHHNs. This small sample size raises the possibility of model overfitting and limited statistical power. To address these issues, we employed Firth penalized logistic regression to obtain more stable and unbiased estimates. Additionally, we performed internal validation using bootstrapping (*n* = 10,000) to confirm the stability of our predictive model. Despite these efforts, the results should be interpreted with caution, and larger multicenter studies are warranted to provide more precise effect estimates. Despite the use of robust statistical methods such as Firth regression and bootstrap validation to improve model stability, the relatively small number of rapid recurrence events (*n* = 21) remains a limitation. The resulting wide confidence intervals suggest that our findings should be interpreted as exploratory. Future multicenter studies with larger cohorts are warranted to confirm the stability and generalizability of the proposed nomogram. Second, histological confirmation was not available for all NHHNs identified on MRI. However, it is essential to note that NHHN is primarily an imaging-based entity defined as a hypointense nodule on the hepatobiliary phase without arterial enhancement. By its nature, the pathological spectrum of these nodules—ranging from premalignant dysplastic nodules to early-stage HCC—cannot be definitively determined without histology, which is seldom feasible in a preoperative clinical setting. Nevertheless, we employed strict, consensus-based imaging criteria to identify these nodules and ensure the reliability of our data. Finally, while we hypothesized the role of surgical stress and the angiogenic switch, we did not evaluate serial change in serum angiogenic factors postoperatively. Future prospective studies incorporating molecular biomarkers are needed to validate the biological link between NHHNs and rapid recurrence.

## 5. Conclusions

In conclusion, while conventional preoperative MRI features associated with MVI and early recurrence did not significantly differentiate patients with rapid recurrence (<6 months) in the entire cohort, the presence of NHHNs and larger tumor size were independently associated with rapid recurrence after curative resection. These findings suggest that NHHN may serve as an imaging marker for identifying patients at risk for particularly rapid recurrence beyond traditional MVI-related features. Recognition of these imaging characteristics may provide additional support for risk stratification in patients undergoing curative resection for HCC.

## Figures and Tables

**Table 1:** Baseline clinical, laboratory, and pathologic characteristics.

Variable	Rapid Recurrence (*n* = 21)	Non-Rapid Recurrence (*n* = 179)	*p*-Value
Age (years)	53.2 ± 8.0	57.5 ± 9.7	0.052
Sex (male/female)			0.151
Male	14 (66.7%)	145 (81.0%)	
Female	7 (33.3%)	34 (19.0%)	
Etiology			0.198
HBV	20	142	
HCV	1	13	
Others (alcohol, cryptogenic)	0	24	
Cirrhosis	14 (66.7%)	108 (60.3%)	0.643
AFP			0.881
≤7.75	10 (47.6%)	89 (49.7%)	
7.75–400	9 (42.9%)	68 (38.0%)	
>400	2 (9.5%)	22 (12.3%)	
PIVKA-II (mAU/mL)			0.007
≤40	5 (23.8%)	102 (57.0%)	
40–400	6 (28.6%)	40 (22.3%)	
>400	10 (47.6%)	37 (20.7%)	
Multiplicity			0.496
Single	17 (81.0%)	156 (87.2%)	
Two	4 (19.0%)	23 (12.8%)	
Microvascular invasion	13 (61.9%)	21 (11.7%)	<0.001
Edmonson–Steiner Grade			0.235
Grade 1	2 (9.5%)	35 (19.6%)	
Grade 2	13 (61.9%)	116 (64.8%)	
Grade 3–4	6 (28.6%)	28 (15.6%)	

Note: Continuous variables are presented as the mean ± standard deviation, while categorical variables are presented as numbers (%).

**Table 2:** Detailed sequence parameters of MRI.

Variables	T1-Weighted IP/OP	HASTE (FS/Non-FS)	DWI	Dynamic T1WI	HBP Imaging (Axial/Coronal)
FOV (mm)	380 × 309	370 × 301/370 × 370	380 × 305	380 × 309	380 × 309
Matrix	256 × 256	384 × 307	192 × 192	384 × 218	221 × 176/320 × 176
TR (ms)	150.0	1400/1500	3100	3.82	3.82
TE (ms)	IP 2.46, OP 1.23	184/216	46.0	1.92	1.92
Echo space (ms)	N/A	4.08	0.66	N/A	N/A
Flip angle (degrees)	70	131	90	9	15
Slice thickness (mm)	5.0	5.0	6.0	3.0	3.0/4.0
Bandwidth (Hz/Px)	1090	723/766	1736	480	510
Echo-train length	N/A	250/307	154	N/A	N/A
Acceleration factor	2	2/3	3	4	4
NSA	1	1	1	1	1
Acquisition time (s)	40	64/77	158	16	15

Note: IP = in phase; OP = opposed phase; HASTE = half-Fourier single-shot turbo spin echo; DWI = diffusion-weighted imaging; HBP = hepatobiliary phase; FOV = field of view; TR = repetition time; TE = echo time; NSA = number of signals averaged.

**Table 3:** Comparison of preoperative MRI features.

Variable	Rapid Recurrence (*n* = 21)	Non-Rapid Recurrence (*n* = 179)	*p*-Value
Tumor size, cm	5.9 ± 4.4	3.1 ± 2.2	<0.001
Multiplicity ≥ 2 tumors	4 (19.0%)	23 (12.8%)	0.496
Non-smooth margin	7 (33.3%)	55 (30.7%)	0.806
Peritumoral enhancement	12 (57.1%)	67 (37.4%)	0.100
Peritumoral hypointensity	6 (28.6%)	37 (20.7%)	0.406
Diffusion restriction	20 (95.2%)	171 (95.5%)	1.000
ADC (×10^−3^ mm^2^/s)	0.93 ± 0.13	0.96 ± 0.11	0.462
NHHN	14 (66.7%)	28 (15.6%)	<0.001

Note: Continuous variables are presented as the mean ± standard deviation, while categorical variables are presented as numbers (%).

**Table 4:** Univariate and multivariate Firth logistic regression analysis of preoperative MRI features for predicting rapid recurrence (<6 months).

Variables	Univariate Analysis		Multivariate Analysis	
	OR (95% CI)	*p*-Value	OR (95% CI)	*p*-Value
Tumor size (per cm)	1.30 (1.13–1.49)	<0.001	1.32 (1.12–1.56)	0.001
Multiplicity	1.60 (0.49–5.16)	0.435		
Non-smooth margin	1.13 (0.43–2.95)	0.807		
Arterial peritumoral enhancement	2.23 (0.89–5.57)	0.086	1.14 (0.39–3.37)	0.810
Peritumoral HBP hypointensity	1.54 (0.56–4.23)	0.407		
Diffusion restriction	0.94 (0.11–7.87)	0.951		
ADC value (×10^−3^ mm^2^/s)	0.21 (0.00–13.26)	0.456		
NHHN	10.79 (4.00–29.11)	<0.001	11.30 (3.75–34.04)	<0.001

## Data Availability

The data presented in this study are available on request from the corresponding author. The data are not publicly available due to institutional privacy and ethical restrictions.

## Abbreviations

The following abbreviations are used in this manuscript:

HCC	Hepatocellular carcinoma
HBP	Hepatobiliary phase
ADC	Apparent diffusion coefficient
NHHN	Non-hypervascular hepatobiliary phase hypointense nodule
IQR	Interquartile range
OR	Odds ratio
MVI	Microvascular invasion
DWI	Diffusion-weighted imaging
CI	Confidence interval
